# Supplementary material for: Differential equation modeling of cell population dynamics in skeletal muscle regeneration from single-cell transcriptomic data
Source: PLoS Comput Biol. 2025 Oct 10;21(10):e1013575. doi: 10.1371/journal.pcbi.1013575 (PMC12533972; doi:10.1371/journal.pcbi.1013575)
Supplement: Attachment — Submitted filename: referee_report_PLOS.pdf [file pcbi.1013575.s003.pdf]

Referee report on "Differential Equation Modeling of Cell Population Dynamics in Skeletal Muscle Regeneration from Single-Cell Transcriptomic Data" by Renad Al-Ghazawi, Xiaojian Shao, Theodore J. Perkins

The authors propose a relatively new model of muscle regeneration, focusing in particular on cell-cell communication between myogenic and immune cell types. The presence of the stem (satellite) cells in two distinct states (quiescent and activated) is another interesting novelty formulated by the model.

Moreover, the authors use two datasets, taken from [33] and [34], to validate the model and tune the parameters characterizing it.

The paper seems to be interesting, from a mathematical and experimental point of view. However, before accepting it for publication, two important issues must be resolved.

The authors assert that the model is similar to the earlier models published in [9, 11, 12], but different in some details, but the differences, in particular with respect to the model described in [12], are not explained, though the two models are based on equations and assumptions which are sometimes completely different.

For example, at p. 9, lines 160/161, the authors speak about "an inflammatory process driven by pro-inflammatory cytokines, growth factors and metabolites", but, differently from [12], these mediators are not included in the equations.

Furthermore, equations (4) and (5), concerning monocytes and macrophages M_1, are completely different from the similar equations in [12] (eqs. (3), (4)). In the latter, the effects of N_d and M_d depend on macrophages M_2. The more M_2 are, the less is the effect of N_d and M_d. Why don't the authors consider these effects, in their model?

Moreover, in equations (5) and (6) there are two nonlinear terms, where the effects of M_d and N_d are completely different from the corresponding (bilinear) terms in [12] (eqs. (4), (5)). In the former, the more are N_d and M_d, the less is the effects of the nonlinear term; in the latter, the more are N_d and M_d, the more is the effect of the bilinear terms. This is a clear and heavy discrepancy between the two models and must be absolutely explained.

Thus, a deeper description of the differences between the models is definitely necessary.

The second part of the paper, devoted to the parameter fitting by means of two scRNA-seq datasets taken from [33] and [34] is very intriguing, giving to the model a great chance to be validated.

However, it is for me a bit disorienting the fact that the data here used lack of any measurement error, which should be naturally included in any experimental

data. Taking into account the errors, the fitting could be much more plausible, allowing the curve to better approximate the data.

Apart from this crucial point, the authors should be much more cautious, when they say, for example, that "simulation and data are in broad, qualitative agreement" (p. 20, line 369), or that "collectively, these results show that the model is capable of producing qualitatively correct trajectories for all cell types" (p. 23, lines 427/428), because, for example, it seems to me that in Fig. 4 the fittings for M_2, QSC, ASC and M_c are absolutely unsatisfactory.

The authors should have to better explain and deepen the discrepancies between experimental data and simulations. For example, speaking about the fitting of M_2 (p. 22, lines 392/398), an analysis of the reason for a very low peak with respect to the experimental data should be absolutely added.

Moreover, I suggest to modify the sentence at p. 26, line 483 in "model that qualitatively captures"

Other issues:

The sentence at p. 26, lines 493/495 ("For one, [...] recorded in experimental data") is absolutely obvious in every fitting of experimental data and is not a peculiar characteristic of this model. Thus I suggest to discard it.

The authors should have to explain what is the meaning of $MAD(\theta)$.

In Fig. 3 caption it must be indicated that the solid lines are related to McKellar data, while dashed lines are related to De Micheli data, because in the plots it is not evident.

Last, but not least, a plan of the paper should be added at the end of the Introduction.

Minor corrections:

1) p. 3, lines 42/43: do the authors mean "which takes into consideration? or describes? or models? the numbers and activities of different cell types over time"?

2) p. 5, lines 91/92: do the authors mean "there has been considerable use"?

3) p. 6, line 99 and line 100: I prefer "previous computational studies on muscle regeneration"

4) p. 7, lines 121/122: write "clean up damaged tissue"

5) p. 8, line 136 and p. 26, line 483: do the authors mean "population dynamics model", as in the title?

6) p. 8, line 152: I prefer "The fitting of the values of these variables"

7) p. 10, line 192: write "is driven by N"

8) p. 13, line 233: write ”differentiate or return to quiescence”

9) p. 14, lines 245/246: similarly to p. 13, line 242 and p. 14, lines 244/245, write ”and M_d to approximately”

10) p. 15, lines 267/268: write ”large numbers of endothelial cells and fibro-adipogenic progenitors (FAPs) are present”

11) along all the paper: the authors should have to indicate always in the same way either McKellar's and De Micheli's data and dataset (see for example p. 18, lines 334, 336 and 348; Table 2; Figure 3 etc.) or McKellar and De Micheli data and dataset (see for example p. 16, lines 300 and 303; p. 18, line 333; p. 19, lines 359/360; Figure 4 etc.)

12) p. 18, line 351: do the authors mean ”by the corresponding scRNA-seq”?

13) p. 19, Table 2, line 2: write ”function of days post injury (DPI)”

14) p. 22, lines 404/405: write ”as a fraction [...] returns to quiescence”.

15) along all the paper: write either ”time-series” (see for example p. 2, line 24 etc.) or ”time series” (see for example p. 2, line 2; p. 23, line 422 etc.)

16) p. 23, lines 417/418: write ”the qualitative shape [...] makes sense”

17) p. 27, line 514: I think that the sentence ”nearly all terms appear linearly” is incorrect: apart from the nonlinear terms, each variable appears in a linear form, but the terms are in the great majority bilinear. Thus I suppose that the authors meant either ”nearly all the variables appear linearly” or ”nearly all the terms appear either linear or bilinear”

18) p. 30, line 571: the authors should have to explain the meaning of the term ’vst’.

19) p. 33, lines 633/634: do the authors mean ”where θ is the vector of unknown parameters to optimize”?

20) p. 33, line 640: write ”The initial conditions [...] were”
