# Supplementary material for: Differential equation modeling of cell population dynamics in skeletal muscle regeneration from single-cell transcriptomic data
Source: PLoS Comput Biol. 2025 Oct 10;21(10):e1013575. doi: 10.1371/journal.pcbi.1013575 (PMC12533972; doi:10.1371/journal.pcbi.1013575)
Supplement: Attachment — Submitted filename: Response.pdf [file pcbi.1013575.s004.pdf]

We thank the reviewers for their insightful feedback on our manuscript. Below we provide detailed responses to each point. The high-level summary of changes is that:

1. We provide more detailed literature-based justifications of our model equations and comparison to previous models. We also eliminated to two terms in our equations (and hence two parameters) that did not contribute substantially to the modeled trajectories.

2. We re-analyzed the scRNA-seq data to allow analysis across biological replicates at each day. This, and other updates to the Seurat pipeline, result in some (but not major) changes to the experimental time-series data. Importantly, it allows us to know the variability in the experimental data.

3. We developed a more uniform approach to calibrating cell proportions to cells per cubic millimeter, using monocyte/macrophage data from a single, coherent study rather than data taken for different cell types from different studies, as in our first submission.

4. We explain in more detail the model simulation and model-fitting approach, including error function, initial parameter estimates, and integration details.

5. We discuss in greater detail limitations of our work and avenues for future research.

Sincerely,

R. Al-Ghazawi, H. Lezzeik, X. Shao, T. J. Perkins

Reviewer #1

The authors propose a relatively new model of muscle regeneration, focusing in particular on cell-cell communication between myogenic and immune cell types. The presence of the stem (satellite) cells in two distinct states (quiescent and activated) is another interesting novelty formulated by the model. Moreover, the authors use two datasets, taken from [33] and [34], to validate the model and tune the parameters characterizing it.

The paper seems to be interesting, from a mathematical and experimental point of view. However, before accepting it for publication, two important issues must be resolved.

The authors assert that the model is similar to the earlier models published in [9, 11, 12], but different in some details, but the differences, in particular with respect to the model described in [12], are not explained, though the two models are based on equations and assumptions which are sometimes completely different.

For example, at p. 9, lines 160/161, the authors speak about "an inflammatory process driven by pro-inflammatory cytokines, growth factors and metabolites", but, differently from [12], these mediators are not included in the equations.

In our work, the signaling molecules that carry information between cells are not modeled explicitly. Rather, we model influences between cell types are direct links in the model, without detailing the sometimes-multiple pathways that convey those influences. Almost all of the cell-cell influences modeled in [12] also follow this strategy, with the exception of the proinflammatory cytokines, which are made explicit in that model. We have added text below each of our model equations to explain how they are similar or different to refs [9,11,12].

Furthermore, equations (4) and (5), concerning monocytes and macrophages M1, are completely different from the similar equations in [12] (eqs. (3), (4)). In the latter, the effects of Nd and Md depend on macrophages M2. The more M2 are, the less is the effect of Nd and Md. Why don't the authors consider these effects, in their model?

In general, our strategy has been to aim for the simplest model possible that allows use to capture key features of regeneration dynamics. In our fitted trajectories, M2 macrophages do not reach appreciable numbers until later in the trajectory. By that time, almost all M-to-M1 conversion has already occurred, and indeed even M1 numbers are also declining, as the M2/anti-inflammatory environment is being established. Hence, we would not expect M2 numbers to play a significant role in influencing M-to-M1 conversion, even if such a term were added to our model.

Moreover, in equations (5) and (6) there are two nonlinear terms, where the effects of Md and Nd are completely different from the corresponding (bilinear) terms in [12] (eqs. (4), (5)). In the former, the more are Nd and Md, the less is the effects of the nonlinear term; in the latter, the more are Nd and Md, the more is the effect of the bilinear terms. This is a clear and heavy discrepancy between the two models and must be absolutely explained. Thus, a deeper description of the differences between the models is definitely necessary.

Indeed, this last point is a significant qualitative difference between our model and previous ODE models. The terms in question were first introduced in [11], citing as justification the review paper Tidball et al. 2010 (doi:10.1152/ajpregu.00735.2009). That paper states that control of the M1 to M2 transition is complex and not well understood, depending for instance on distinctions between lysed, apoptotic and necrotic neutrophils -- neutrophil fate distinctions that neither our model nor refs [9,11,12] consider. Moreover, those observations were specific to neutrophils, not dead myonuclei.

Regardless, a key point from Tidball et al. is that it is the phagocytosis of [dead] neutrophils or possibly other dead material by M1 macrophages that promotes the transition to M2 phenotype. In [11], the rate of M1 to M2 transition is highest when the instantaneous rate of phagocytosis is highest, namely when dead material and M1's are both highly abundant. However, rather than considering the instantaneous rate of phagocytosis, we propose that the more phagocytosis that has taken place, the more the transition to M2 will be favored. More phagocytosis having occurred means that fewer dead neutrophils or myonuclei remain, because they have been consumed. So, if the extent of phagocytosis having occurred positively relates to M1-to-M2 transition, then the extent of dead cells remaining should negatively relate to the M1-to-M2 transition, consistent with our equation. This also makes logical sense, because the more dead cells there are in an environment, the more need there is for M1 macrophages to consume them. It would not make sense if M1 macrophages disappeared faster when the need for their phagocytic services was greater. This reasoning is also found in the recent agent-based model by Haase et al. 2024 (https://doi.org/10.7554/eLife.9192), where the tendency for an M1 macrophage to transition to an M2 state depends on the amount of "debris" the cell has already phagocytosed.

The second part of the paper, devoted to the parameter fitting by means of two scRNA-seq datasets taken from [33] and [34] is very intriguing, giving to the model a great chance to be validated.

However, it is for me a bit disorienting the fact that the data here used lack of any measurement error, which should be naturally included in any experimental data. Taking into account the errors, the fitting could be much more plausible, allowing the curve to better approximate the data.

We have completely re-analyzed the scRNA-seq data for this revision, and as part of that re-analysis, we have kept track of replicate-to-replicate variability in the amounts of the different cell types. That variability is now visually displayed in Figure 4, so that one can better reason about which parts of the data are more certain. We experimented with the possibility of weighing the error at each point inversely to the standard deviation, as is sometimes done in regression analysis. (And indeed this can be justified within a maximum likelihood framework under certain assumptions.) However, because some data points had substantially higher standard deviations than others, we found that the effect of such a weighting was to

nearly eliminate some data points from the error function, which we found undesirable. So, we settled on informing the reader of the certainty of the data points by adding standard deviation bars, but not altering the error function itself.

Apart from this crucial point, the authors should be much more cautious, when they say, for example, that "simulation and data are in broad, qualitative agreement" (p. 20, line 369), or that "collectively, these results show that the model is capable of producing qualitatively correct trajectories for all cell types" (p. 23, lines 427/428), because, for example, it seems to me that in Fig. 4 the fittings for M2; QSC; ASC and Mc are absolutely unsatisfactory. The authors should have to better explain and deepen the discrepancies between experimental data and simulations. For example, speaking about the fitting of M2 (p. 22, lines 392/398), an analysis of the reason for a very low peak with respect to the experimental data should be absolutely added.

Moreover, I suggest to modify the sentence at p. 26, line 483 in "model that qualitatively captures"

These precise sentences are no longer in the manuscript. There remain places where we talk about "qualitative" match with the data. But, in such cases we are much more explicit with what we mean by qualitative agreement, discussing details such as: the time at which a cell type peaks in our simulations versus the data (and prior literature), the height of that peak compared to the data, the rapidity in rise or fall of a cell type's numbers, or even the degree to which the two datasets McKellar and De Micheli agree or disagree on these features. Moreover, we are more forthright in criticizing some of the shortfalls of our model trajectories.

Other issues:

The sentence at p. 26, lines 493/495 ("For one, [...] recorded in experimental data") is absolutely obvious in every fitting of experimental data and is not a peculiar characteristic of this model. Thus I suggest to discard it.

That paragraph has been removed.

The authors should have to explain what is the meaning of MAD(θ).

The error function is now explained clearly in section 2.5 after line 367.

In Fig. 3 caption it must be indicated that the solid lines are related to McKellar data, while dashed lines are related to De Micheli data, because in the plots it is not evident.

This figure has changed substantially to display the individual replicates. In addition, a legend was added in the upper left explaining the meaning of the lines and dots in the plots.

Last, but not least, a plan of the paper should be added at the end of the Introduction.

We have added a paragraph to the end of the Introduction section outlining the logic and contributions of the different Results sections.

Minor corrections:

1) p. 3, lines 42/43: do the authors mean "which takes into consideration? Or describes? or models? the numbers and activities of different cell types over time"?

Changed "explains" to "describes".

2) p. 5, lines 91/92: do the authors mean "there has been considerable use"?

Yes, corrected.

3) p. 6, line 99 and line 100: I prefer "previous computational studies on muscle regeneration"

Corrected.

4) p. 7, lines 121/122: write "clean up damaged tissue"

Corrected.

5) p. 8, line 136 and p. 26, line 483: do the authors mean "population dynamics model", as in the title?

Corrected.

6) p. 8, line 152: I prefer "The fitting of the values of these variables"

We felt the sentence was confusing, so deleted it.

7) p. 10, line 192: write "is driven by N"

Corrected.

Reviewer #2: This manuscript uses a very interesting approach of modelling cell population dynamics in skeletal muscle injury using single-cell RNA sequencing data. It is encouraging to see that the authors carefully considered the main cell types involved in the injury process and how cell composition evolves over time after injury. However, there are some major points that need to be addressed:

1. The two datasets used in the study contain libraries from multiple mice, yet this was not considered in the analysis or presented in the results.

In our revised analysis, we report separate cell type counts and proportions for each replicate. In the model-fitting figure, we present empirical data points as means and standard deviations across the replicates. This allows the reader to understand the confidence in the individual data points, when we compare the two datasets to each other, and when we compare to the model simulations. We did not alter the error function to account for uncertainty in the data, as we found this had undesirable effects on the model trajectories.

2. While the model has considered number and rate change of each cell type, this set up does not account for the fact that the injury in tissue is not homogenous and distribution of each cell type would depend on microenvironment, information that single cell RNA sequencing data just does not provide.

This has been noted in the Discussion as a limitation to our study. Our model, which describes regeneration in a cubic millimeter of tissue, could potentially be extended to a spatio-temporal model, but this is beyond the scope of the present study. In addition, we suggest in the Discussion the use of spatial transcriptomics as a potential data source for modeling spatial heterogeneity in cell type distributions.

3. In Table S2 and Table S3, the number of each cell type should be provided to show if the study is powered to model so many cell types.

The supplementary data has been revised to include: cell type numbers for every day and replicate of both McKellar and De Micheli datasets, cell proportions for every day and replicate, per-day means and variances of proportions across replicates, and calibrated per-cubic-millimeter cell counts.

4. Line 357 to 360: the description of initial parameter setting using “by hand”, “rough reasoning”, and “by-eye” is not what to be expected in a scientific publication.

This has been replaced with order-of-magnitude estimates, using systematic reasoning described in the manuscript.

Reviewer #3: The manuscript presents a computational model of skeletal muscle regeneration, integrating single-cell RNA sequencing (scRNA-seq) data to capture the dynamic interactions between satellite cells, immune cells, and other key players in muscle repair. The study describes the phases of muscle regeneration, emphasizing the roles of M1 and M2 macrophages in modulating satellite cell activation, proliferation, and differentiation. The paper is well-written and can be of interest for the readership of the journal.

General Comment:
The manuscript addresses a relevant topic—the cross-talk between different components of the system at the molecular level to facilitate muscle regeneration. One major challenge of studying cross-talk at the molecular level is the difficulty in tracking real-time dynamics of molecules and proteins, which may result in an incomplete picture of physiological processes. In contrast, studies at the macroscopic level, such as those analyzing muscle-muscle and muscle-organ interactions under a Network Physiology approach, explore physiological coordination/cross-talk across different temporal scales. Studying organ interactions at multiple levels and developing new analytical approaches to bridge the gap between molecular and macroscopic scales will provide a more comprehensive understanding of physiological function. Given that organ interactions play a crucial role at all spatial levels, it would be valuable to briefly discuss this perspective in your discussion section to help position your findings within this broader framework.

See below some references related to this point—please note that the authors are not required to cite these works, as they are provided solely for informational purposes on this topic: doi.org/10.3389/fnetp.2021.711778; doi.org/10.1113/JP286963

Discussion points have been added talking about spatial scale (including organ- and organism-level influences) and temporal scale, including aging. References have been added, and suggestions for future work augmented.

Minor Comments

See below some other minor comments that may help strengthen the manuscript:

- The model primarily represents regeneration in young, healthy muscle, yet aging significantly impacts satellite cell function, immune response, and fibrosis. Incorporating an aging-related perspective, even if speculative (future research), would strengthen the manuscript’s broader applicability.

The McKellar data was performed on 20-month old mice, which are considered old. The De Micheli data was performed on 3-7 month old mice, which are considered adult. For most cell types, we did not observe large differences between the two datasets. Nevertheless, aging is of great interest, and does influence regeneration, as numerous publications attest. Modeling such changes in regeneration with aging has been added to the future work plans.

- The model assumes a uniform muscle environment, but oxygen availability, inflammatory signals, and mechanical stress vary across the injury site, influencing regeneration. Suggestion: Acknowledge this limitation and mention future potential refinements.

This limitation has been acknowledged and suggested as an avenue for future work.

- Satellite cell activation and immune function are highly energy-dependent. A brief mention of how metabolic factors (e.g., oxidative stress, glucose availability) may affect regeneration would provide additional biological context.

We have added mention of metabolism and disease as factors affecting satellite function, and potential avenues for future modeling work.
