# Supplementary material for: Differential equation modeling of cell population dynamics in skeletal muscle regeneration from single-cell transcriptomic data
Source: PLoS Comput Biol. 2025 Oct 10;21(10):e1013575. doi: 10.1371/journal.pcbi.1013575 (PMC12533972; doi:10.1371/journal.pcbi.1013575)
Supplement: Attachment — Submitted filename: second_report_PLOS.pdf [file pcbi.1013575.s005.pdf]

**Second referee report on "Differential Equation Modeling of Cell Population Dynamics in Skeletal Muscle Regeneration from Single-Cell Transcriptomic Data" by Renad Al-Ghazawi, Xiaojian Shao, Theodore J. Perkins**

The authors highly improved the paper, following the referees' comments.

Unfortunately the new parts added to the manuscript contain several minor misprints that should be corrected, before the publication.

**Minor corrections:**

**1)** p. 9, line 167; p. 19, line 367; p. 32, line 630: write "Python", instead of "python"

**2)** p. 14, line 271: I prefer "Unlike previous works"

**3)** p. 16, line 296: do the authors mean "the same cell types"?

**4)** p. 17, line 317: write "who does not have"

**5)** p. 17, line 326: do the authors mean "of that lineage at day zero"?

**6)** p. 17, line 327: if the replicates are more than two, please, write "There is high variability among replicates"

**7)** pp. 18/19, lines 360/361: what do the authors mean with "per-replicate proportion"?

**8)** p. 19, line 372: write "at day $d$."

**9)** p. 20, lines 390/391: I prefer "is on the order of 10,000"

**10)** p. 20, line 395: write "Nelder-Mead algorithm"

**11)** p. 22, line 406: write "does not produce a reasonable fit"

**12)** p. 22, line 416; p. 23, line 436: write "was not fitted"

**13)** p. 23, line 433: I prefer "We could no doubt increase"

**14)** p. 24, line 458: do the authors mean "enough cells to differentiate"?

**15)** p. 24, line 460: write "is consistent with experimentally"

**16)** p. 25, line 479: do the authors mean "sensitive to multiples of the system parameters"?

**17)** p. 25, line 488: write "Conversely"

**18)** p. 26, line 493: I prefer "to all of them is given by the neutrophile"

**19)** p. 29, line 572: write "an important step"

**20)** p. 30, line 583: I prefer "the possibility to capture and explore"

**21)** p. 32, line 629: write "the system of ODEs"

**22)** p. 33, line 643: I prefer "Each matrix component"
