# Supplementary material for: Differential equation modeling of cell population dynamics in skeletal muscle regeneration from single-cell transcriptomic data
Source: PLoS Comput Biol. 2025 Oct 10;21(10):e1013575. doi: 10.1371/journal.pcbi.1013575 (PMC12533972; doi:10.1371/journal.pcbi.1013575)
Supplement: Attachment — Submitted filename: Response_auresp_2.pdf [file pcbi.1013575.s006.pdf]

Dear Editor,

We thank the editor and reviewers for their feedback on our revised manuscript. We have implemented the grammatical corrections suggested by reviewer 1, fixed the order of grant funding statements (on the online form), and added descriptive legends after the References section for the two supplementary files.

Sincerely,

R. Al-Ghazawi, H. Lezzeik, X. Shao, T. J. Perkins
